# Ischemic Preconditioning of Rat Livers from Non-Heart-Beating Donors Decreases Parenchymal Cell Killing and Increases Graft Survival after Transplantation

**DOI:** 10.1155/2012/236406

**Published:** 2012-07-19

**Authors:** Robert T. Currin, Xing-Xi Peng, John J. Lemasters

**Affiliations:** ^1^Department of Cell & Developmental Biology, University of North Carolina, Chapel Hill, NC 27599, USA; ^2^Center for Cell Death, Injury & Regeneration, Departments of Pharmaceutical & Biomedical Sciences and Biochemistry & Molecular Biology, Medical University of South Carolina, Charleston, SC 29425, USA

## Abstract

A critical shortage of donors exists for liver transplantation, which non-heart-beating cadaver donors could help ease. This study evaluated ischemic preconditioning to improve graft viability after non-heart-beating liver donation in rats. Ischemic preconditioning was performed by clamping the portal vein and hepatic artery for 10 min followed by unclamping for 5 min. Subsequently, the aorta was cross-clamped for up to 120 min. After 2 h of storage, livers were either transplanted or perfused with warm buffer containing trypan blue. Aortic clamping for 60 and 120 min prior to liver harvest markedly decreased 30-day graft survival from 100% without aortic clamping to 50% and 0%, respectively, which ischemic preconditioning restored to 100 and 50%. After 60 min of aortic clamping, loss of viability of parenchymal and nonparenchymal cells was 22.6 and 5.6%, respectively, which preconditioning decreased to 3.0 and 1.5%. Cold storage after aortic clamping further increased parenchymal and non-parenchymal cell killing to 40.4 and 10.1%, respectively, which ischemic preconditioning decreased to 12.4 and 1.8%. In conclusion, ischemic preconditioning markedly decreased cell killing after subsequent sustained warm ischemia. Most importantly, ischemic preconditioning restored 100% graft survival of livers harvested from non-heart-beating donors after 60 min of aortic clamping.

## 1. Introduction

Liver transplantation surgery is a viable alternative for patients with end-stage liver disease but the number of heart-beating cadavers suitable for liver donation remains a key limitation. In human kidney transplantation, organ donation from non-heart-beating cadavers is now employed successfully at many centers [1]. Organ donors are typically terminally ill patients who do not meet the criteria of brain death and whose life support is withdrawn at the request of the family. After cardiac arrest occurs and death is pronounced several minutes later, the organs are harvested.

The use of livers from non-heart-beating donors is also emerging as an important stratagem to expand the liver donor pool [2]. Organs from non-heart-beating cadaver donors typically experience several minutes of warm ischemia prior to cold preservation. Warm ischemic injury that occurs to livers after cardiac arrest can severely compromise graft viability. Early clinical results with livers from non-heart-beating donors were poor, and two-month graft survival was only 50% even for donors that were extubated in an operating room setting [3]. With more rapid organ harvesting, clinical outcomes have improved, but rates of primary nonfunction, initial poor function, and ischemic-type biliary strictures remain greater than with donor livers from heat-beating cadaver donors [2]. Consequently, new and different strategies are needed to block warm ischemic injury in this context and to improve the outcome of non-heart-beating cadaver donation in liver transplantation.

Ischemic conditioning is the application of brief episodes of nonlethal ischemia and reperfusion to confer protection against sustained ischemia, which is showing therapeutic potential in various clinical settings [4, 5]. In rodent studies, ischemic preconditioning of the liver protects against injury after subsequent prolonged warm ischemia, particularly in fatty livers [6–8]. Decreases of transaminase release and sinusoidal endothelial cell killing also occur after cold preservation, which improve graft survival after orthotopic transplantation [9, 10]. In human liver surgery, ischemic preconditioning decreases postoperative transaminases and hepatic apoptosis, particularly in patients with mild-to-moderate steatosis, but in liver transplantation the benefit of ischemic preconditioning remains to be conclusively established [11–14]. The effect of ischemic preconditioning on graft injury and survival after transplantation of livers from non-heart-beating donors is not well studied. Here, we show that preconditioning with 10 min of warm hepatic ischemia markedly decreases hepatocellular and endothelial cell killing after subsequent sustained warm ischemia and after sustained warm ischemia followed by cold storage. Most importantly, ischemic preconditioning restores graft survival of livers harvested from non-heart-beating donors.

## 2. Methods

### 2.1. Orthotopic Rat Liver Transplantation

All animal protocols conformed to criteria of the Institutional Animal Care and Use Committee. Orthotopic rat liver transplantation was performed in male Lewis rats (220–280 g) under ether anesthesia using an arterialized two-cuff method by slight modification of the procedure of Steffen et al. [15]. For the donor operation, the liver was freed from its peritoneal attachments, and the common bile duct was cannulated with a polyethylene tube and divided. Cold University of Wisconsin (UW) solution (Viaspan, Dupont Pharma, Wilmington, DE) was infused through the portal vein. The suprahepatic inferior cava, subhepatic inferior cava, portal vein, and celiac artery were divided at the level of the diaphragm, left renal vein, splenic vein, and splenic artery, respectively. The liver was excised and placed in a bath of ice-chilled UW solution. Cuffs were then placed on the portal vein and subhepatic inferior cava before storage at 0–1°C in an ice water bath.

In recipient rats, the proper hepatic and gastroduodenal arteries were divided at their origin, leaving a stump of the common hepatic artery. The stump was clamped at the base of the dissected segment. The bifurcation of the proper hepatic and gastroduodenal arteries was cut, leaving a funnel-shaped opening to which a cuff was attached. After dividing the bile duct at the hilum, the suprahepatic inferior cava, portal vein, and subhepatic inferior cava were clamped and divided, and the recipient liver was removed. The donor liver was then rinsed with 10 mL of Ringer's solution at 37°C. Subsequently, the suprahepatic inferior cava was anastomosed with a running suture, and the portal vein, subhepatic inferior cava, and hepatic artery were connected in sequence by insertion of cuffs. The bile duct was anastomosed over an intraluminal polyethylene splint.

### 2.2. Ischemic Preconditioning and Aortic Clamping

To induce ischemic preconditioning, the abdomen was opened under ether anesthesia, and the hepatic artery and portal vein were occluded with a mini-bulldog vascular clamp for 10 min. The clamp was then released, and the liver was reperfused for 5 min prior to harvesting. To simulate non-heart-beating organ donation, the thorax was opened under ether anesthesia, and the ascending aorta clamped for 5 to 120 min, followed immediately by harvest of the liver.

### 2.3. Cell Killing

To assess cell viability after storage, livers were reperfused for 15 min with Krebs-Henseleit bicarbonate buffer (KHB) containing 500 *μ*M trypan blue at an initial flow rate of 5 mL/min increasing to 30 mL/min over the first 5 min. In some experiments, stored livers were reperfused for 5 min with ice-cold UW solution containing 500 *μ*M trypan blue at an initial flow rate of 5 mL/min increasing to 15 mL/min after 5 min [16, 17]. After trypan blue infusion, livers were fixed by perfusion for 2 min with 2% paraformaldehyde in 0.1 M NaPi buffer, pH 7.4. The temperature of the fixation was the same as the preceding perfusion. After this initial fixation, the left lateral lobe was cut into 1 cm slices and placed in ice-cold fixative. The tissue was then embedded in paraffin, sectioned, and stained with eosin or hematoxylin/eosin. For each liver, nuclear trypan blue uptake in parenchymal and nonparenchymal cells was determined in 5 random periportal and pericentral regions in eosin-stained sections using a 60X objective and expressed as the percentage of total nuclei counted in hematoxylin/eosin stained sections.

### 2.4. Statistics

Differences in survival were analyzed using Fisher's exact test, and differences between means were analyzed by analysis of variance (ANOVA). One-tailed tests were used to test unidirectional hypotheses, for example, that ischemic preconditioning decreases storage/reperfusion injury and improves graft survival. Group sizes are given in the legends to the figures. Errors represent the standard error of the mean. *P* values of less than 0.05 were considered to be significant.

## 3. Results

### 3.1. Decreased Graft Survival after Warm Ischemia Prior to Liver Harvest

**Figure 1:**
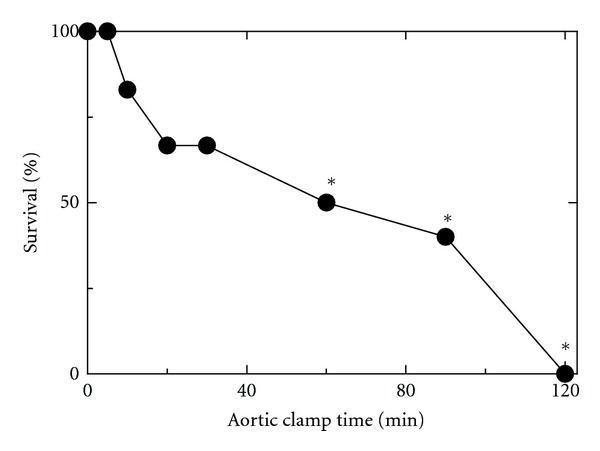
Loss of graft survival after aortic clamping. Aortas were clamped for 0 to 120 min. After clamping, livers were flushed with ice-cold UW solution. After 2 h of cold storage, the livers were transplanted into recipient rats, as described in section 2. Data are from 5 to 12 transplantations per time point. *P < 0.05 compared to 0 min clamping by Fisher's exact test.

Harvesting livers from non-heart-beating cadaver donors represents a potential means to expand the donor pool for human clinical liver transplantation. Accordingly, we harvested rat livers after various periods of aortic clamping and transplanted them into syngeneic recipient Lewis rats. When the aorta was not cross-clamped prior to liver harvest, 30-day graft survival was 100% after 2 h of cold storage (Figure 1). Graft survival was also 100% after 5 min of aortic clamping. After 10 min and longer, graft survival decreased progressively from 83% after 10 min to 50% after 60 min and 0% after 120 min. Thus, the livers tolerated only a very short period of warm ischemia before graft survival after transplantation began to decline. Necropsies revealed that none of the deaths was due to an obvious surgical error, such as a leaky arterial or venous anastomosis.

### 3.2. Loss of Parenchymal and Nonparenchymal Cell Viability after Warm Ischemia and Warm Ischemia Followed by Cold Storage

In another set of experiments, we assessed what cell types were losing viability as a consequence of various periods of warm ischemia. Aortas were cross-clamped, and after various periods of time, cold UW solution containing trypan blue was infused for 5 min followed by perfusion fixation with cold phosphate-buffered paraformaldehyde. Viable cells exclude trypan blue, whereas nonviable cells take up trypan blue into their nuclei. Thus, by counting trypan blue positive nuclei in eosin-counterstained histological sections, loss of viability of both parenchymal and nonparenchymal cells was determined.

**Figure 2:**
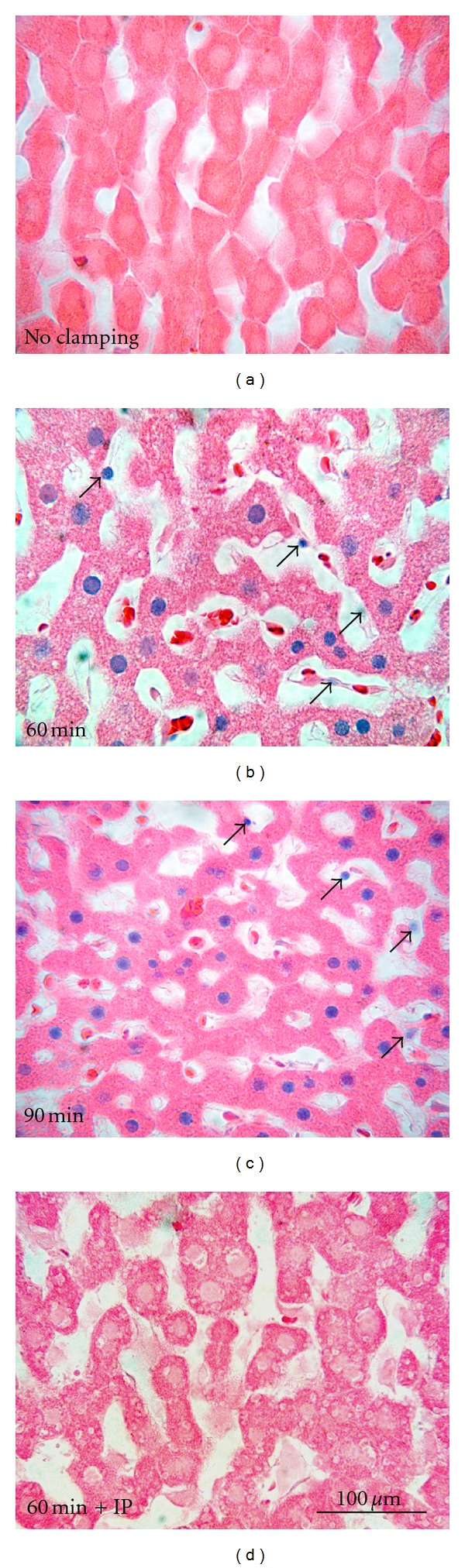
Light microscopy of livers after aortic clamping. In (a, b, c), the aortas of anesthetized rats were clamped for 0, 60, and 90 min, respectively, and their livers were then infused with cold UW solution containing trypan blue to label nonviable cells, as described in section 2. In d, a liver was first subjected to 10 min of ischemia followed by 5 min of reperfusion before 60 min aortic clamping. Trypan blue was then infused, as described for (a–c). Blue nuclei in eosin-counterstained sections represent mostly nonviable parenchymal cells. Arrows identify examples of nonviable nonparenchymal cells.

**Figure 3:**
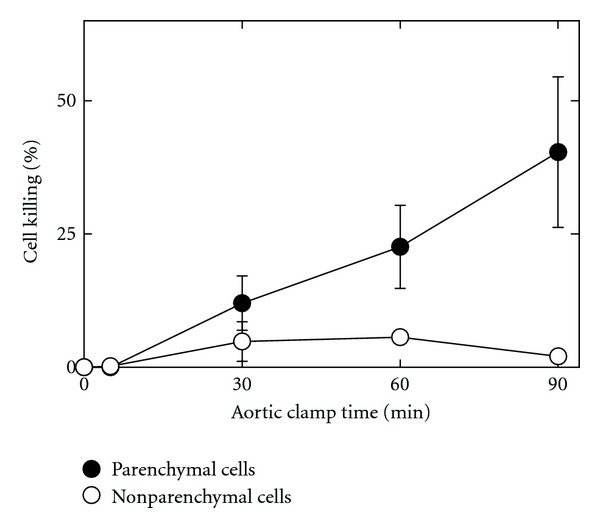
Increased parenchymal and nonparenchymal cell killing after aortic clamping. Aortas were clamped for 0 to 90 min. After clamping, livers were immediately infused with cold UW solution containing trypan blue and fixed, as described in Figure 2. Data represent means ± S.E.M. from 3 to 5 rats per group per time point.

Without aortic cross-clamping, trypan blue-stained nuclei were extremely rare in histological sections (Figure 2(a)). By contrast, with aortic cross-clamping, trypan blue labeling progressively increased (Figures 2(b) and 2(c) and Figure 3). Most trypan blue labeling occurred in hepatic parenchymal cells (hepatocytes), but some nonparenchymal cells also began to label after longer periods of clamping (Figures 2(b) and 2(c), arrowheads). The percentage of trypan blue-labeled parenchymal and nonparenchymal nuclei was averaged for several livers subjected to various times of aortic cross-clamping. After 0 and 5 min of aortic cross-clamping, trypan blue labeling of parenchymal or nonparenchymal cells was less than 0.5% (Figure 3). After longer times of cross-clamping, parenchymal cell killing (loss of viability) steadily increased from 12% after 30 min to 40% after 90 min. Nonparenchymal cell killing increased to a lesser extent to a maximum of 6% after 60 min. Nonparenchymal killing appeared to decrease after 90 min, but this finding may be artifactual due to masking of nonparenchymal labeling by parenchymal nuclear labeling or to washout of nuclei of nonviable nonparenchymal cells as the livers were infused with trypan blue-containing UW solution.

**Figure 4:**
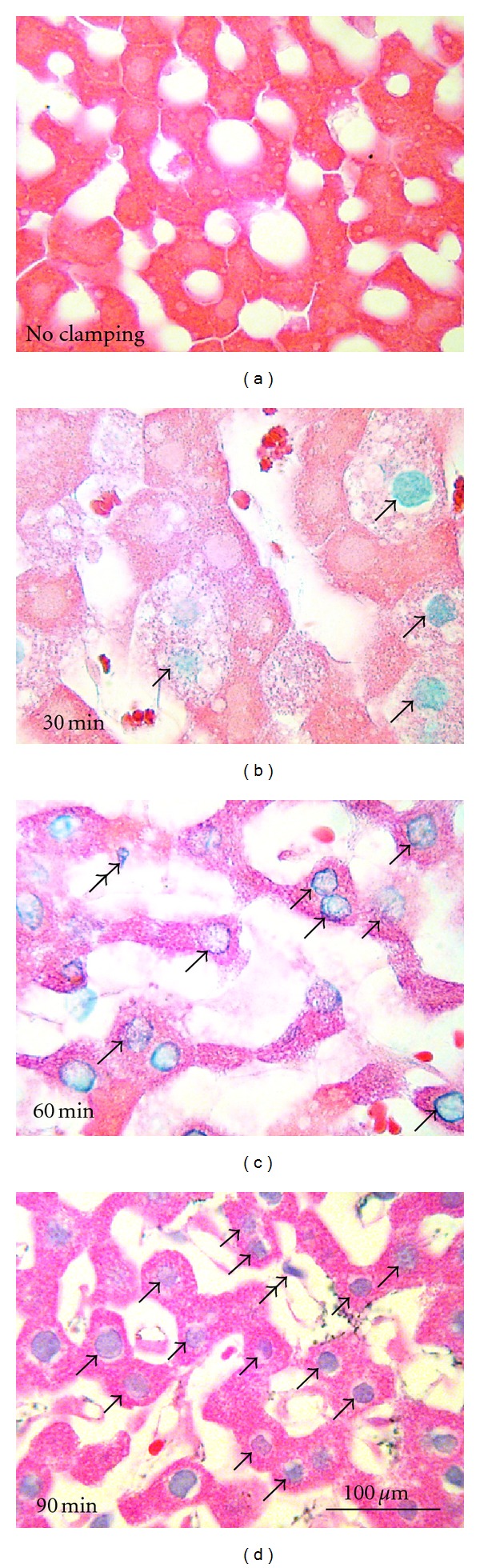
Light microscopy of livers after aortic clamping and cold storage/reperfusion. Aortas were clamped for 0 to 90 min. After clamping, livers were stored for 2 h in cold UW solution, followed by 15 min of warm reperfusion with KHB containing trypan blue to label nonviable cells, as described in section 2. Blue nuclei in eosin-counterstained sections represent nonviable parenchymal and nonparenchymal cells, as illustrated by arrows and double arrows respectively.

**Figure 5:**
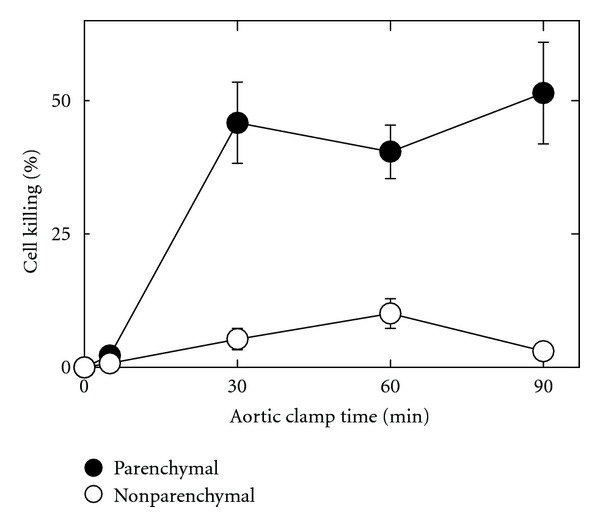
Parenchymal and nonparenchymal cell killing after aortic clamping and cold storage/reperfusion. Aortas were clamped for 0 to 90 min, and livers were cold stored and reperfused, as described in Figure 4. Data represent means ± S.E.M. from 5 rats per group per time point.

To assess the additional effect of cold storage and reperfusion, aortas were cross-clamped for 0 to 90 min, and the livers were then infused with cold UW solution and stored for 2 h. After storage, livers were reperfused at 37°C for 15 min with KHB containing trypan blue and fixed for histology. Cold storage and reperfusion in the absence of aortic clamping led to virtually no trypan blue staining of either parenchymal or nonparenchymal cells (Figure 4(a)). With increasing times of aortic clamping followed by cold storage and reperfusion, parenchymal cell killing increased markedly (Figures 4(b), 4(c), and 5). After 30 and 60 min of cross-clamping and cold storage, parenchymal cell killing was 3.8 and 1.8 times that observed after clamping but no cold storage (Figure 5 compared to Figure 3, *P* < 0.05). After 90 min of cross-clamping, cold storage caused 28% more parenchymal cell killing than in the absence of cold storage, but this difference was not statistically significant (Figure 4(d) and Figure 5). None of the differences between nonparenchymal cell killing after clamping alone and nonparenchymal cell killing after clamping plus storage were statistically significant.

### 3.3. Improved Survival after Ischemic Preconditioning of Liver Grafts Subjected to Warm Ischemia and Cold Storage

**Figure 6:**
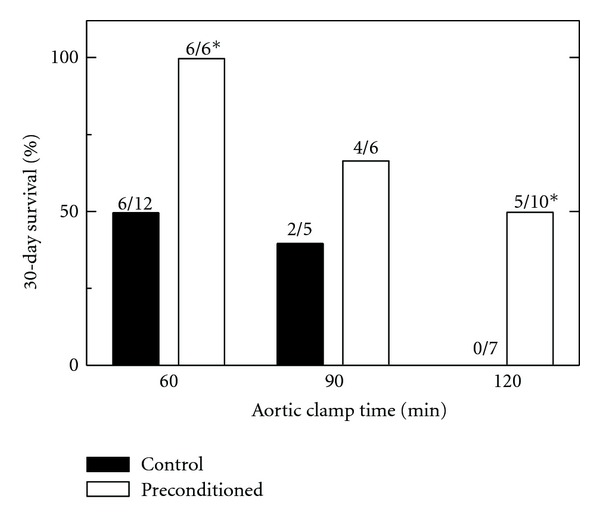
Improved graft survival by ischemic preconditioning before aortic clamping. Anesthetized rats were given a sham operation (control) or ischemic preconditioning by clamping of the hepatic artery and portal vein for 10 min followed by 5 min of blood reperfusion (preconditioned). The aortas of the rats were then clamped for 60, 90, or 120 min. After clamping, livers were infused with cold UW solution, and stored for 2 h. After storage, livers were transplanted into recipient rats, and recipient survival after 30 days was determined. Numbers above the bars indicate survivors/total transplants. *P < 0.05 versus control by Fisher's exact test.

In an effort to make donor livers resistant to the deleterious effects of aortic clamping prior to organ harvest and cold storage, we performed an ischemic preconditioning protocol whereby the hepatic artery and portal vein were occluded for 10 min. Subsequently, the vascular clamp was removed to allow the reflow of blood to the liver. After 5 more min, the aorta was cross-clamped for 60 to 120 min, and the livers were harvested, stored 2 h in UW solution, and transplanted. In comparison to non-preconditioned liver grafts, 30-day survival of liver grafts subjected to ischemic preconditioning increased from 50 to 100% after 60 min of aortic clamping (*P* < 0.05), 40 to 67% after 90 min of clamping (*P* = 0.39), and 0 to 50% after 120 minute of clamping (*P* < 0.05) (Figure 6). When it occurred, graft failure developed relatively rapidly. After clamp times of 60, 90 and 120 min, survival of animals that did not live to 30 days averaged 2.4, 2.5, and 4.8 days, respectively, without preconditioning. With preconditioning, average time to death of animals not surviving 30 days was 3.0 and 4.8 days after 90, and 120 min of aortic clamping.

### 3.4. Decreased Parenchymal and Nonparenchymal Cell Killing by Ischemic Preconditioning in Livers Subjected to Warm Ischemia

**Figure 7:**
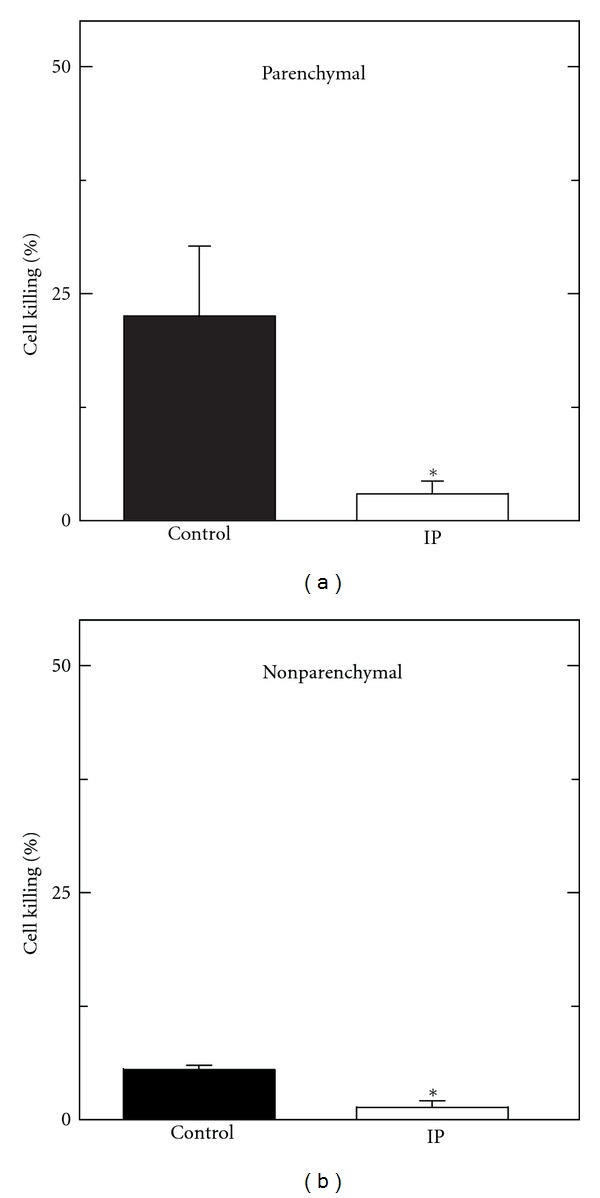
Decreased parenchymal and nonparenchymal cell killing by ischemic preconditioning after aortic clamping. Anesthesized rats were given a sham operation (control) or ischemic preconditioning by clamping of the hepatic artery and portal vein for 10 min followed by 5 min of blood reperfusion (IP). The aortas of the rats were then clamped for 60 min. After clamping, the livers were infused with cold UW solution containing trypan blue to label nonviable parenchymal (a) and nonparenchymal (b) cells. Data represent means ± S.E.M. from 5 rats per group per time point. *P < 0.05 versus control by ANOVA.

Since ischemic preconditioning improved survival of liver grafts subjected to warm ischemia before storage, we investigated how ischemic preconditioning influenced cell killing during these treatments. Livers were subjected to ischemic preconditioning or a sham operation. Subsequently, aortic clamping was imposed for 60 min, and the livers were infused with cold trypan blue-containing UW solution followed by fixation for histology. Our goal was to determine how ischemic preconditioning affected parenchymal and nonparenchymal cell killing prior to cold storage. As shown in Figure 7, ischemic preconditioning (IP) decreased parenchymal (a) and nonparenchymal (b) cell killing determined by trypan blue labeling to 3.0 to 1.5%, respectively, from 22.5% and 5.6 % after sham treatment (control) (*P* < 0.05).

**Figure 8:**
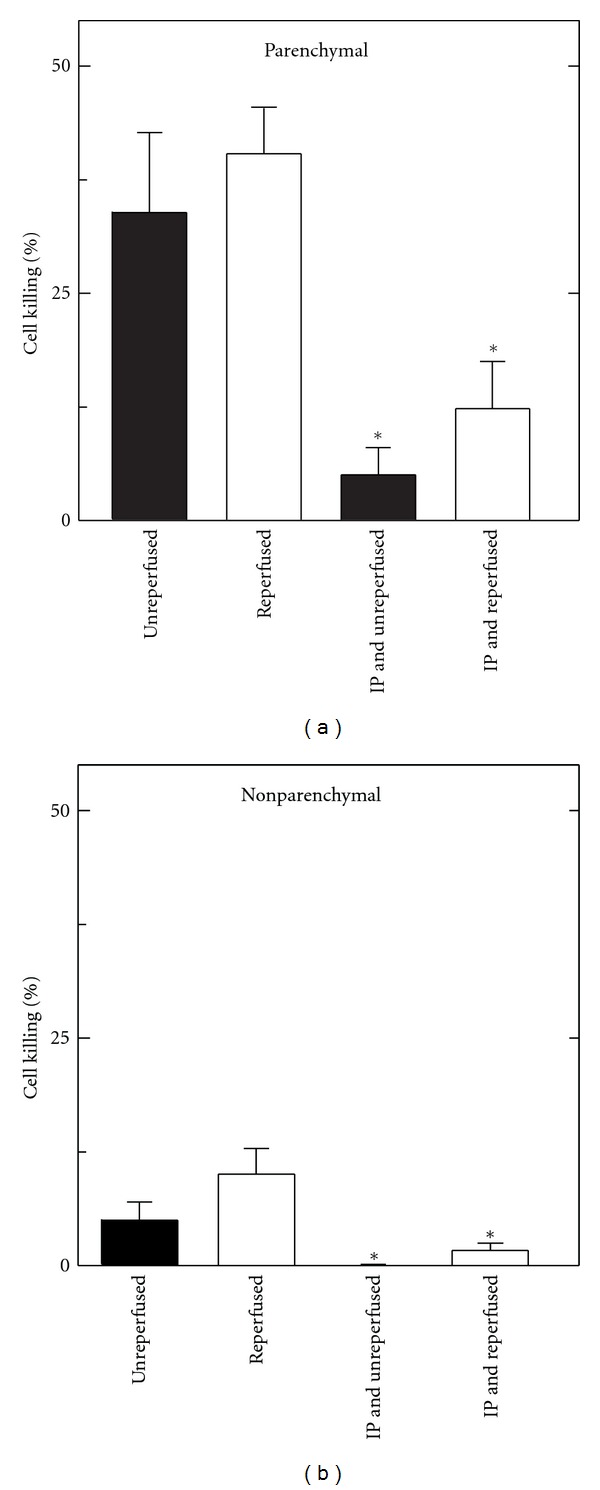
Decreased parenchymal and nonparenchymal cell killing by ischemic preconditioning after aortic clamping and cold storage with and without reperfusion. Anesthesized rats were given a sham operation or ischemic preconditioning (IP) by clamping of the hepatic artery and portal vein for 10 min followed by 5 min of blood reperfusion. The aortas of the rats were then clamped for 60 min. After clamping, the livers were stored for 2 h in cold UW solution. Subsequently, the livers were infused for 5 min with cold UW solution containing trypan blue to label nonviable cells in the absence of warm reperfusion (unreperfused) or infused for 15 min with warm KHB containing trypan blue to label nonviable cells after warm reperfusion (reperfused), as described in section 2. Nonviable parenchymal cells (a) and nonparenchymal cells (b) were counted from 5 livers per group. *P < 0.05 compared to the corresponding non-IP group by ANOVA.

Similarly, we subjected livers to ischemic preconditioning (IP) or sham treatment followed by 1 h of aortic clamping and 2 h of cold storage in UW solution. At the end of storage, the livers were either flushed with cold trypan blue-containing UW solution to assess cell killing at the end of storage (unreperfused) or reperfused with warm trypan blue-containing KHB for 15 min to assess cell killing after reperfusion (reperfused). As shown in Figure 8, parenchymal (left panel) and nonparenchymal (right panel) cell killing without preconditioning was about the same in stored livers that were not reperfused as in those that were reperfused. By contrast, ischemic preconditioning prior to aortic clamping and cold storage caused large and statistically significant decreases of both parenchymal and nonparenchymal cell killing measured at the end of cold storage with and without reperfusion.

## 4. Discussion

The success of liver transplantation surgery for patients with end-stage liver disease has caused growth worldwide of waiting lists for liver transplantation surgery, which greatly outnumber the available donor livers. The use of livers from non-heart-beating donors is one potential source for increasing the organ donation pool. Non-heart-beating donors have become an important source of kidney donations and are becoming an increasing source for liver donation [1, 2]. However unlike kidney grafts, liver grafts must recover function much more quickly and appear to be more susceptible to harvest, preservation, and reperfusion injury. 

In our non-heart-beating model, the aortas of Lewis rats were cross-clamped for a specified period of time, stored for 2 h in UW solution, and transplanted. After as little as 10 min of aortic clamping, graft survival decreased after orthotopic rat liver transplantation (Figure 1). With increasing aortic clamp time, there was a continued decline in graft survival to 50% after 60 min of aortic clamping and 0% after 120 min of warm ischemia.

To better understand the injury caused by aortic clamping, we infused trypan blue at the end of aortic clamping to label nonviable cells. To minimize the effects of reperfusion we infused cold UW solution containing trypan blue for 5 min. Trypan blue labeling indicated that cellular killing was predominately to parenchymal cells (Figure 2). Trypan blue nuclear labeling signifies onset of necrotic cell death. Although not analyzed in detail, apoptosis was largely absent as shown by the lack of nuclear condensation, lobulation, and chromatin aggregation, which is consistent with earlier studies of both warm and cold ischemia (reviewed in [18]). Parenchymal cell killing started after as little as 10 min of aortic clamping and steadily increased over time (Figure 3). Parenchymal cell killing increased further after subsequent cold storage in UW solution and reperfusion, but no significant differences in nonparenchymal cell killing were observed between aortic clamping and aortic clamping plus cold storage and reperfusion (Figures 4 and 5). Unlike cold ischemia [19, 20], warm ischemia involved principally parenchymal cells. Injury to parenchymal cells began to increase after 10 min of aortic clamping and continued to increase over time, which correlated with transplantation studies where graft failure also began to occur and progressively increased after 10 min or more of aortic clamping.

In heart and other organs including liver, preconditioning by short periods of ischemia followed by reperfusion protects against longer periods of ischemia [4]. Our model of ischemic preconditioning entailed clamping the hepatic artery and portal vein for 10 min, followed by 5 min of blood reperfusion. Such ischemic preconditioning markedly increased graft survival after 60 and 120 min of aortic cross-clamping (Figure 6). Substantial survival after as much as 120 min of aortic cross-clamping illustrates the robustness of protection by ischemic preconditioning. Likewise, ischemic preconditioning decreased parenchymal and nonparenchymal cell killing after aortic clamping and after aortic clamping followed by cold storage plus reperfusion (Figures 7 and 8). In some models, repetitive (up to 3) intervals of short ischemia/reperfusion further improve protection by ischemic preconditioning [4, 5]. Future studies will be needed to optimize preconditioning in terms of ischemia time and number of repetitions for non-heart-beating liver donation, although use of repetitions of ischemia/reperfusion may be impractical in a clinical donor situation. Ischemic-type biliary strictures can develop 1 to 4 months after liver transplantation whose incidence increases with non-heart-beating donation [2, 21, 22]. Future studies will also be needed to determine if ischemic preconditioning can also decrease the incidence of such strictures.

The mechanisms of action for ischemic preconditioning of the liver involve a variety of factors and pathways, including adenosine, nitric oxide, and activation of protein kinases (e.g., phosphatidylinositol 3-kinase, protein kinase C, p38 MAP kinase) and transcription factors (e.g., signal transducer and activator of transcription 3, nuclear factor-kB and hypoxia-inducible factor 1) [5]. In the context of cold storage/reperfusion injury to liver, we and others showed in a rat model of orthotopic liver transplantation that ischemic preconditioning improves survival of liver grafts harvested from heart-beating rat donors [10, 23, 24]. Improved graft survival is associated with decreased sinusoidal endothelial cell killing and Kupffer activation mediated at least in part by an adenosine A_2_ receptor pathway coupled to increased cAMP [9]. Ischemic preconditioning also decreases hepatic injury, attenuates mitochondrial dysfunction, reduces free radical formation, and improves regeneration of small-for-size liver grafts, possibly by increasing mitochondrial superoxide dismutase expression [25]. Increasingly, ischemic preconditioning is being employed clinically to decrease hepatic injury after liver transplantation and other surgeries [12].

By contrast to the predominantly nonparenchymal cell injury that occurs after cold storage/reperfusion, the findings of the present study show that warm ischemia induced by aortic clamping caused loss of viability predominantly to parenchymal cells, namely, the hepatocytes. This lethal parenchymal cell injury then led to graft failure after orthotopic rat liver transplantation. Reperfusion was not a key factor contributing to cell killing after aortic clamping, since cell death could be shown in the absence of warm reperfusion by direct infusion of cold UW solution containing trypan blue. Nonetheless, subsequent brief cold storage and warm reperfusion did increase cell killing moderately. The change in cell type and role of reperfusion in hepatic cell killing after warm versus cold ischemia suggests different mechanisms of injury. Since parenchymal cell injury after aortic clamping occurred before cold storage, assessment of the suitability of donor livers from non-heart-beating cadavers might be possible before cold storage by examining trypan blue labeling in biopsies.

In conclusion, ischemic preconditioning protected strongly against parenchymal cell killing after aortic clamping and markedly improved survival of grafts from non-heart-beating donors. In a clinical setting, uncontrolled episodes of hypoxia and hypoperfusion may contribute to protective preconditioning, but organ manipulations such as ischemic preconditioning are currently prohibited prior to declaration of donor death. However, future changes in living wills and accepted ethical practices may permit use of ischemic preconditioning in terminally ill donors just prior to withdrawal of life support. Moreover, better understanding of the mechanisms underlying protection by ischemic preconditioning in the specific context of non-heart-beating liver donation may permit use of pharmacological strategies to simulate ischemic preconditioning. In this way, ischemic preconditioning or its pharmacological surrogate might one day be applied clinically in advance of liver donation after planned removal of life-sustaining treatment such as mechanical ventilation.
